# Cardiorespiratory coupling in the bottlenose dolphin (*Tursiops truncatus*)

**DOI:** 10.3389/fphys.2023.1234432

**Published:** 2023-09-21

**Authors:** A. Fahlman, J. C. Mcknight, A. M. Blawas, N. West, A. G. Torrente, K. Aoki

**Affiliations:** ^1^ Fundación Oceanografic de la Comunidad Valenciana, Gran Vía Marques del Turia 19, Valencia, Spain; ^2^ Kolmården Wildlife Park, Kolmården, Sweden; ^3^ Global Diving Research SL, Valencia, Spain; ^4^ Sea Mammal Research Unit, Scotland, United Kingdom; ^5^ Duke University Marine Laboratory, Nicholas School of the Environment Duke University, Beaufort, NC, United States; ^6^ Dolphin Quest, Kahala Resort, Waikoloa, HI, United States; ^7^ Institut de Génomique Fonctionnelle, Université de Montpellier, CNRS, INSERM, Montpellier, France; ^8^ Department of Marine Bioscience, Atmosphere and OceanResearch Institute, The University of Tokyo, Chiba, Japan

**Keywords:** cardiorespiratory physiology, marine mammal, cetacean, heart rate, perfusion, ventilatory tachycardia

## Abstract

**Introduction:** The bottlenose dolphin (*Tursiops truncatus*) is an intermittent breather, where the breath begins with an exhalation followed by inhalation and an extended inter-breath interval ranging from 10 to 40 s. Breathing has been shown to alter both the instantaneous heart rate (i*f*_H_) and stroke volume (iSV) in the bottlenose dolphin, with a transitory ventilatory tachycardia following the breath, and an exponential decrease to a stable i*f*_H_ around 40 beats • min^−1^ during the inter-breath period. As the total breath duration in the dolphin is around 1 s, it is not possible to assess the contribution of exhalation and inhalation to these changes in cardiac function during normal breathing.

**Methods:** In the current study, we evaluated the i*f*_H_ response by separating expiration and inspiration of a breath, which allowed us to distinguish their respective contribution to the changes in i*f*_H_. We studied 3 individual male bottlenose dolphins trained to hold their breath between the different respiratory phases (expiration and inhalation).

**Results:** Our data show that inspiration causes an increase in i*f*_H_, while expiration appears to result in a decrease in i*f*_H_.

**Discussion:** These data provide improved understanding of the cardiorespiratory coupling in dolphins, and show how both exhalation and inhalation alters i*f*_H_.

## Introduction

Cardiorespiratory coupling, where breathing results in variation in the instantaneous heart rate (i*f*_H_), has been reported in seals and cetaceans (Irving, 1939; Ridgway, 1972; Kooyman, 1985; Kaczmarek et al., 2018; Fahlman et al., 2020b; Blawas et al., 2021b). In the bottlenose dolphin (*Tursiops truncatus*), the intermittent breathing pattern begins with a rapid exhalation followed by inhalation with a breath duration of approximately 1 s (Fahlman et al., 2017). The end of the breath, i.e., end-inspiration, results in an initial increase in i*f*_H_ followed by a slow decline to a heart rate between 35 and 50 beats • min^−1^ (Cauture et al., 2019; Fahlman et al., 2020b; Blawas et al., 2021a). During the inter-breath period there is often a sinusoidal variation in i*f*_H_ with a period of varying duration of between 4 and 10 s (Cauture et al., 2019; Blawas et al., 2021a). The magnitude of this respiratory tachycardia varies both with changes in breathing frequency (*f*_R_) and tidal volume (*V*_T_) (Cauture et al., 2019; Blawas et al., 2021a; Blawas et al., 2021b). However, the respiratory characteristics of the bottlenose dolphin, with high respiratory flow during exhalation and a breath duration that lasts one or two heartbeat cycles, makes it difficult to separately evaluate how exhalation and inhalation affect cardiac function.

To improve our understanding of the ventilatory tachycardia, and to assess the variation in i*f*_H_ during the exhalation and inspiration phases in bottlenose dolphins, we measured electrocardiograms (ECGs) using a data logger with suction cup-embedded electrodes and respiratory flow using a custom-built pneumotachometer during both normal breaths and breaths where exhalation and inhalation were separated. Our results provide evidence that normal breaths result in a respiratory tachycardia, and that exhalation and inhalation, respectively, result in a decrease and increase in i*f*_H_. Thus, the cardiac variability in bottlenose dolphins associated with respiration occurs at their typical breathing frequencies between 0.025 and 0.1 Hz. The results also highlight a second frequency of cardiac variability during breathing in the range of 0.1–0.2 Hz, possibly associated with blood pressure regulation. The data presented in the current study imply that both exhalation and inhalation alter i*f*_H_, and that breathing frequency alters cardiac function and heart rate.

## Materials and methods

### Animal information

Three male bottlenose dolphins housed in managed care were studied for this work (Table 1). Individual animal ID, body mass, and year of birth are summarized in Table 1. Dolphins were not restrained and could refuse to participate or withdraw at any point during the experimental trial. Before data collection, animals had been desensitized to the equipment and trained for novel research-associated behaviours using operant conditioning (Fahlman et al., 2015; Fahlman et al., 2020b). These individual animals had participated in similar cardiorespiratory trials on numerous occasions since 2015 (Fahlman et al., 2015; Fahlman et al., 2019a; Fahlman et al., 2019b; Cauture et al., 2019; Fahlman et al., 2020b; Blawas et al., 2021a).

**TABLE 1:** Animal identification (ID), body mass (*M*_*b*_), year of birth (YOB), number of breaths analyzed (N), and mean instantaneous heart rate (*f*_*H*_) for pre- and post-breaths, with either complete breaths (*Full*, exhalation followed by immediate inhalation), or either exhalation (*Ex*) or inhalation (*In*). The uneven number between the number of exhalations and inhalations was due to exhalation followed by inhalations outside the pneumotachometer (i.e., flow not measured, 63H4 and 90N6), or repeated shallow exhalations before a full inspiration (9FL3).

ID	*M*_*b*_ (kg)	YOB	N			Pre-*f*_H_ (beats • min^-1^)			Post-*f*_H_ (beats • min^-1^)		
			*Full*	*Ex*	*In*	*Full*	*Ex*	*In*	*Full*	*Ex*	*In*
63H4	178	1991	8	5	6	79.5 ± 9.0	78.9 ± 9.2	55.3 ± 13.0	88.7 ± 7.1	54.6 ± 8.6	79.8 ± 14.6
9FL3	182	1997	20	39	5	59.2 ± 8.8	62.1 ± 8.5	50.1 ± 14.4	71.9 ± 6.7	44.1 ± 5.7	67.2 ± 6.9
90N6	244	1985	17	9	8	73.4 ± 19.9	62.8 ± 15.9	54.7 ± 5.2	84.4 ± 8.6	55.5 ± 11.2	69.9 ± 2.6

### Research trials

During each trial, the dolphin remained stationary in the water, allowing placement of the ECG data logger on the chest (ECG400-DT, Little Leonardo co.) and placement of the pneumotachometer over the blow-hole (Fahlman et al., 2015; Aoki et al., 2021).

The dolphins performed two types of trials for data collection. During *normal breathing*, an exhalation was followed by an immediate inhalation. During an *exhale hold,* a single exhalation was followed by an inter-breath interval of between 5 and 45 s, followed by inhalation. In one dolphin, several exhalation signals were given, which resulted in repeated exhalations before inhalation, i.e., *repeated exhale hold*. These breathing manoeuvres were repeated during a single session and multiple sessions were repeated for each animal over several days. One individual was trained to perform repeated exhales before inhalation with a respiratory pause between 3 and 5 s.

The dolphins were not fasted, but where inactive before, for between 2 and 5 min, and during each trial, which lasted between 5 and 10 min. All trials were conducted between 7 and 10 November 2022, either in the morning or afternoon.

### Measurements

We analyzed the i*f*_H_ 1–3 s before (pre) and after (post) a full breath (*Full*), exhalation (*Ex*), or inhalation (*In*). We did this to assess the immediate cardiovascular response caused by ventilation, and due to limitations in obtaining extended exhalation holds. The relative change in i*f*_H_ (%) before and after each respiratory phase was analyzed as (post-pre) • pre^−1^ • 100. Thus, a positive or negative change is, respectively, an increase or decrease in i*f*_H_ following a breathing phase.

The respiratory timing was measured using a custom-made pneumotachometer, as previously detailed (Fahlman et al., 2015; Fahlman et al., 2020a), but briefly summarized below. Respiratory flows were measured using a custom-made Fleisch type pneumotachometer (Mellow Design, Valencia, Spain), which housed a low-resistance laminar flow matrix (Item #Z9A887-2, Merriam Process Technologies, Cleveland, OH). A differential pressure transducer (Spirometer Pod, ML 311, ADInstruments, Colorado Springs, CO) was connected to the pneumotachometer with two, 310 cm lengths of 2 mm I.D., firm walled, flexible tubing. The differential pressure transducer was connected to a data acquisition system (Powerlab 8/35, ADInstruments, Colorado Springs, CO), and the data were captured at 400 Hz and displayed on a computer running LabChart (v. 8.1, ADInstruments, Colorado Springs, CO). The respiratory flow was used to determine the beginning and end of each exhalation and inhalation for a total of 117 respiratory events (Table 1). The beginning and end of a respiratory phase were used as time = 0 for the averaging of the heart rate. For example, for a *Full* breath time = 0 for the pre data was at the beginning of the exhalation, while for post time = 0 was the end of the inhalation.

The respiratory flow and heart rate signals were synchronized by aligning start and end times of data collection.

### Statistical analysis

We used a general linear mixed-effects (GLM) model with nested random effects of individual to account for differences between animals. Models were fitted in the R statistical computing software (R Core Team, 2021; RStudio Team, 2021) using the *nlme* package (Pinheiro et al., 2021).

## Results

**FIGURE 1:**
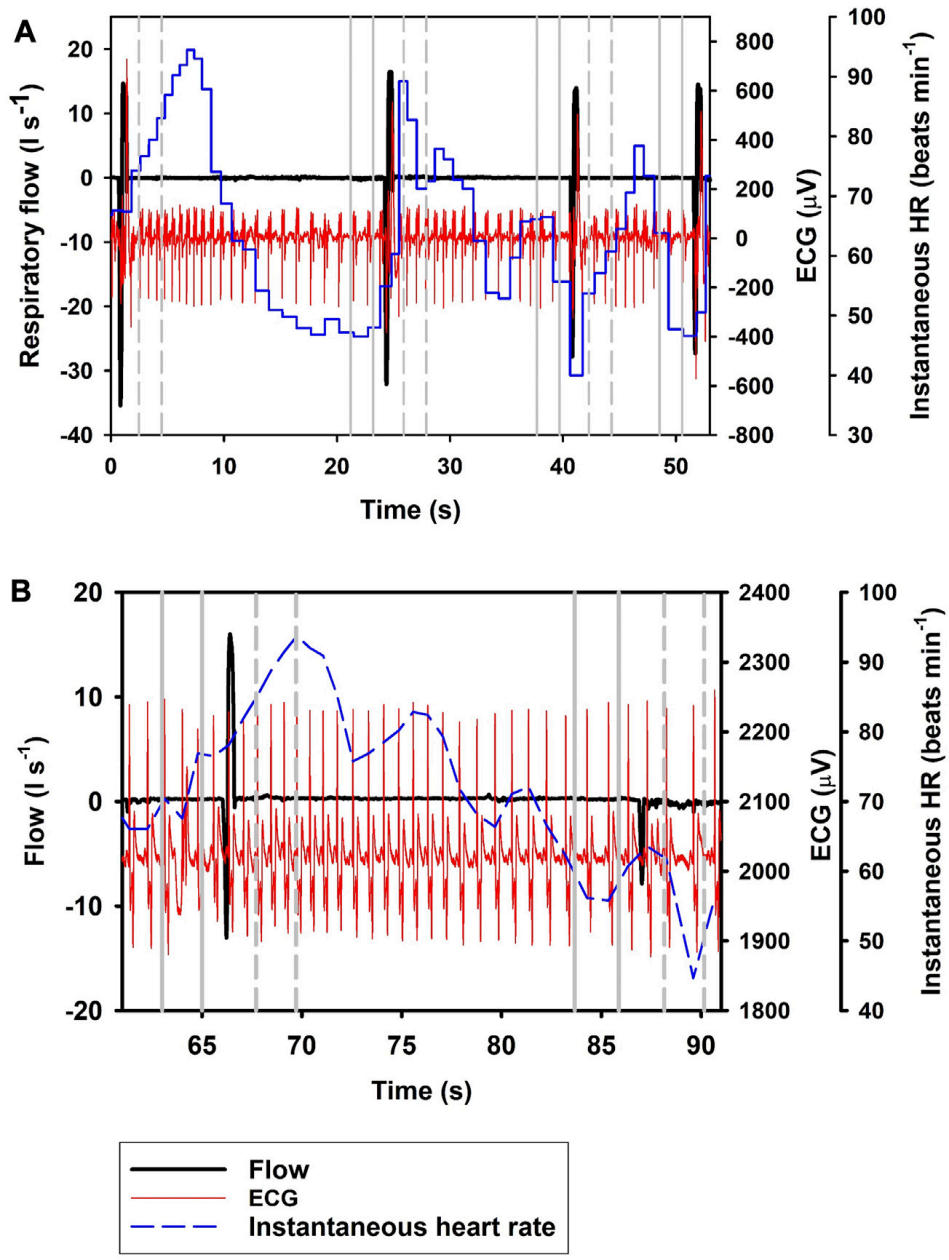
Respiratory flow, raw ECG, and instantaneous heart rate (HR) during **(A)** four single full (*Full*; t_1_ = 0.7 s, t_2_ = 25 s, t_3_ = 41 s, t_4_ = 52 s) breaths, and **(B)** a single full breath (t = 67 s) with a 20 s interbeath interval until the next exhalation (*Ex*; t = 88 s). Vertical lines show section used for analysis, with solid gray line indicating pre- and broken gray line post-breath.

**FIGURE 2:**
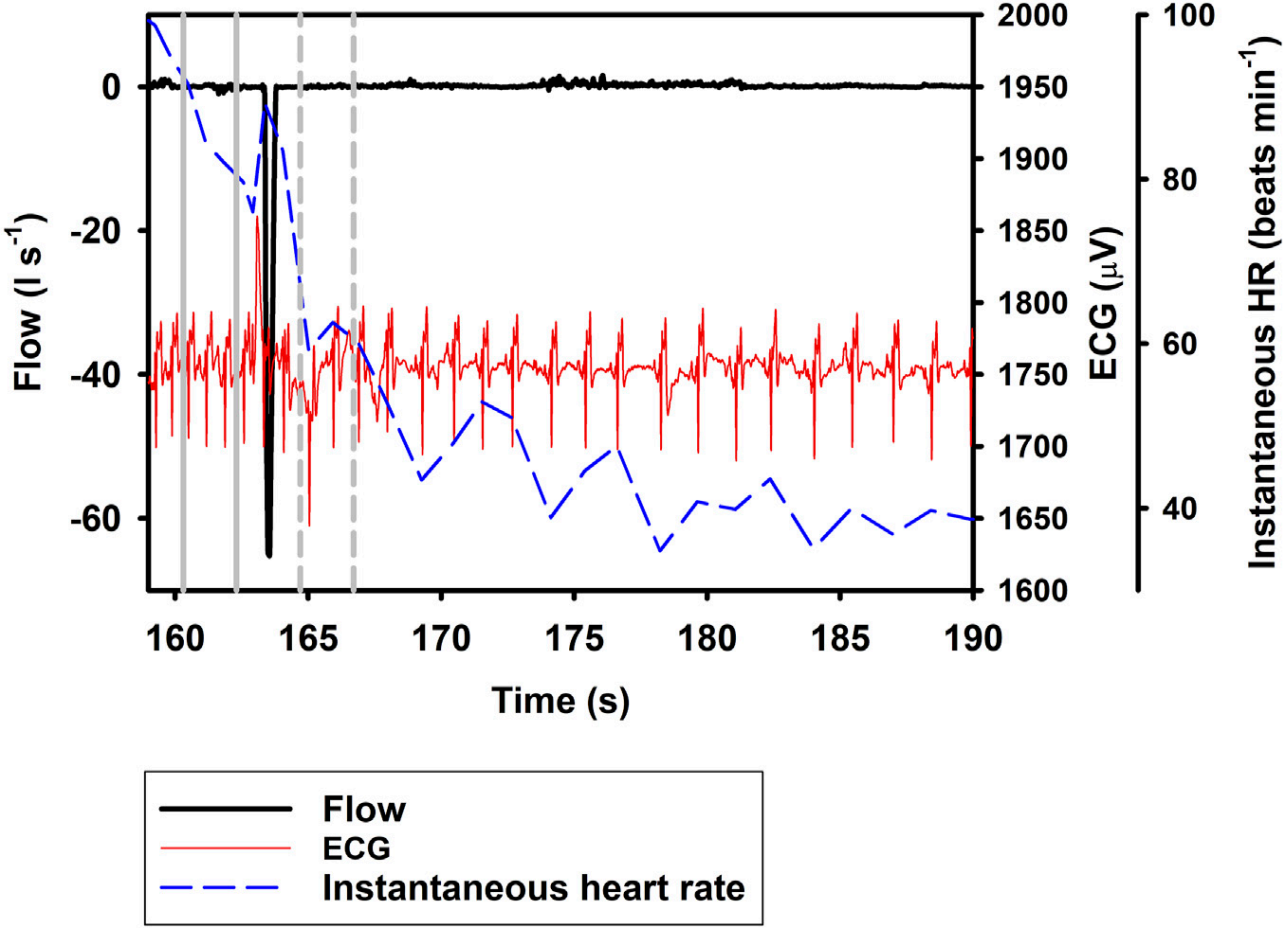
Respiratory flow, raw ECG, and instantaneous heart rate (HR) during a single exhalation (*Ex*; t = 164 s). Vertical lines show section used for analysis, with solid gray line indicating pre- and broken gray line post-breath.

**FIGURE 3:**
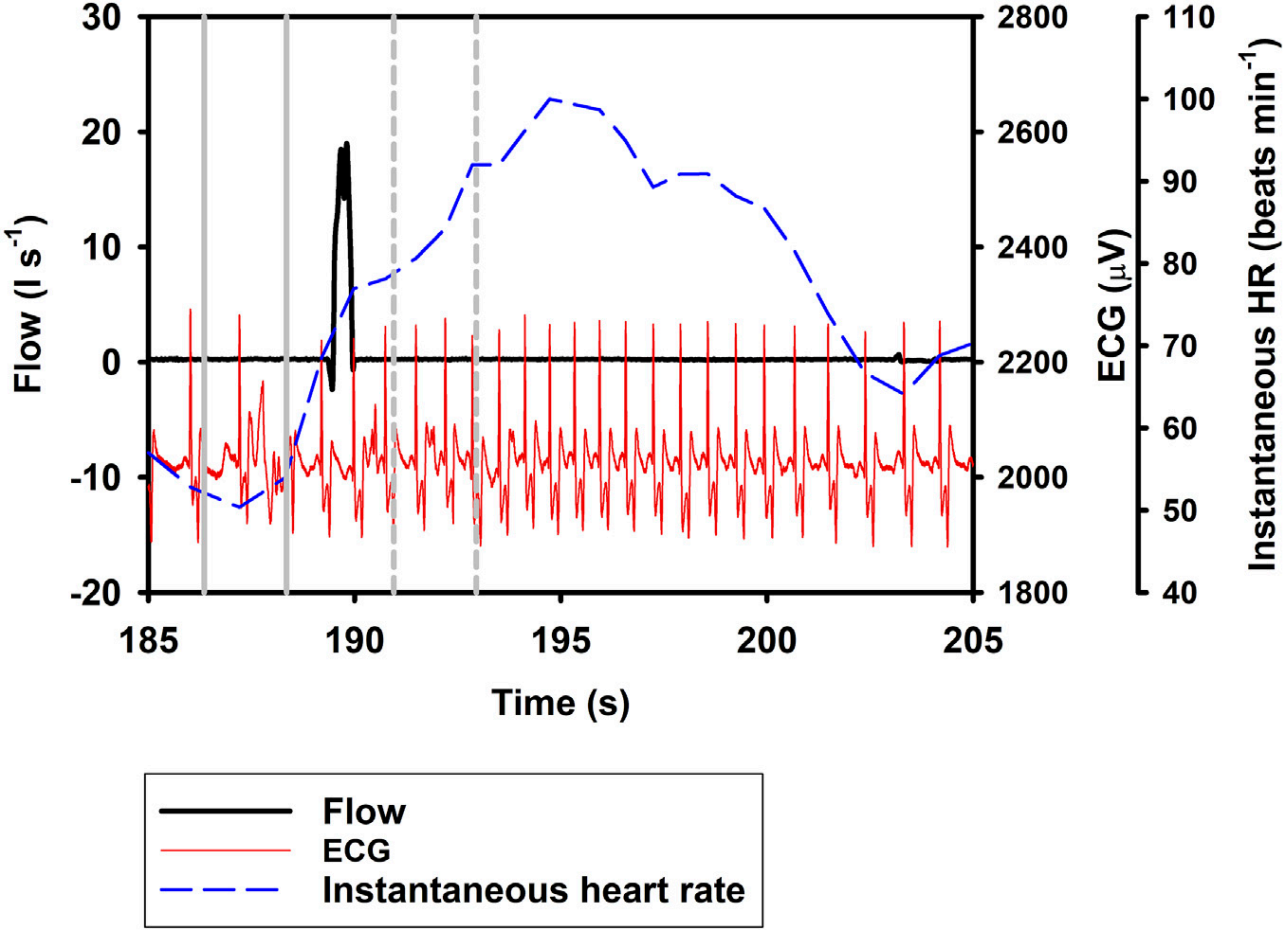
Respiratory flow, raw ECG, and instantaneous heart rate (HR) during a single inhalation (*In*; t = 190 s). Vertical lines show section used for analysis, with solid gray line indicating pre- and broken gray line post-breath.

Figure 1 shows the i*f*_H_ following *Full* breaths, with panel A) showing the response during repeated *Full* breaths, and panel B) following a single *Full* breath followed by an exhalation. The most parsimonious model showed that i*f*_H_ increased by 19.9% following a *Full* breath (Eq. 1; Table 1; Figure 1, df = 2, χ^2^ = 84.2). When breaths were separated into exhalation and inhalation, *Ex* resulted in a 33.5% decrease in i*f*_H_. *In* resulted in an additional 20.1% increase in i*f*_H_ (Eq. 1; Table 1; Figures 2, 3), similar to what we recorded during a normal breathing cycle.

$$\Delta f_{H}\;\left(\%\right)=19.9\;\left(2.8\right)\;–\;33.5\;\left(3.9\right)\;•\;Ex+20.1\;\left(5.5\right)\;•\;In$$ (1)

For repeated *Ex*, the decrease in i*f*_H_ was not affected by the number of repeated exhalations (df = 1, Loglikelihood = 447.4, χ^2^ = 0.03).

## Discussion

In the current study, we show a respiratory tachycardia associated with normal breaths in the bottlenose dolphin, similar to what has been previously reported in this species and also other small to medium sized cetaceans (Irving, 1939; Ridgway, 1972; Fahlman et al., 2020b; Blawas et al., 2021b). We also report that when exhalation and inhalation are separated, the changes in i*f*_H_ associated with exhalation and inhalation appear to occur in opposite directions. In addition, we confirm that the sinusoidal variation in i*f*_H_ of between 0.1 and 0.2 Hz that is present following *Full* breaths (Figure 1B) (Cauture et al., 2019; Fahlman et al., 2020a; Blawas et al., 2021a; Blawas et al., 2021b), also is present after exhalations and inhalations (Figures 2, 3). We propose that this respiratory tachycardia may be important to enhance gas exchange during inspiration and help reduce cardiac work during the extended inter-breath intervals in intermittent breathers. The respiratory tachycardia we report in the bottlenose dolphin has implications for how to define resting heart rate, which may help improve comparative studies.

The data provided in the current study show that following a *Full* breath, there is a temporary 19.9% increase in i*f*_H_ (Figure 1; Eq. 1; Table 1). We restricted the analysis of i*f*_H_ to a 2-s interval between 1 and 3 s before or after the breathing phase for two reasons; first, we wanted to show that the response develops soon following a breath phase, and second, to be able to assess the effect of repeated exhalation. Under conditions of held phases of respiration, our data further suggest a decrease in i*f*_H_ by 33.5% following exhalation (Figures 1, 2), with a 20.1% increase during inhalation (Figure 3; Eq. 1; Table 1). Notably, the magnitude of increase in i*f*_H_ during the short period following a *Full* breath (19.9%, range: 14.9%–59.8%) was less than has been reported in previous studies in cetaceans where the increase can be as high as 100% (Ridgway, 1972; Fahlman et al., 2019a; Cauture et al., 2019; Blawas et al., 2021a; Blawas et al., 2021b). One reason for this could be the variation in the temporal response during *Full* breaths, where the maximal i*f*_H_ following the breath can take several seconds to develop (Figure 1A). Thus, the increase may not have fully developed during our restricted period of analysis following a *Full* breath. When the *In* was separated from the *Ex* phase, it resulted in an additional 20.1% increase in i*f*_H_ (Figure 3, Table 1, Eq. 1). One possible explanation for this result could be that during a normal breath the *Ex* phase partially suppresses the effects of the *In* phase, which gives rise to the temporary increase in i*f*_H_ that we report. However, when breathing phases are separated the responses represent the distinct effect of the *In* and *Ex* phases. Thus, separating the breathing phases allows the effect of lung inflation and deflation on i*f*_H_ to be observed on an extended time scale. (Eq. 1).

Following a *Full* breath in the bottlenose dolphin, the transient increase followed by an exponential decrease in i*f*_H_ between breaths commonly does not reach a stable value until after 7–12 s, but can be as long as 15 s (Figure 1A) (Irving, 1939; Ridgway, 1972; Fahlman et al., 2019b; Cauture et al., 2019; Blawas et al., 2021a; Blawas et al., 2021b). In the present study, when the exhalation was separated from the inhalation, the i*f*_H_ decreased by 33.5% within the 1–3 s after the exhalation. One alternative explanation to the decrease in i*f*_H_ that we observed is that this merely represents the progressive sinusoidal decline during the inter-breath period (Figure 1B). Thus, it is possible that the decrease in i*f*_H_ following exhalation alone is the result of this continuous decline. If so, *Ex* may not result in a further decrease in heart rate, however, we do not believe this is the case as studies in other mammals show a decrease in heart rate during a reduction in lung volume (Hayano et al., 1996; Looga, 1997; Mortola et al., 2015). Finally, in a limited number of samples (n = 64), we analyzed the change in i*f*_H_ between 1 and 5 s following the inhalation or exhalation (i.e., a 4-s interval), which showed no further increase in i*f*_H_ compared to i*f*_H_ between 1 to 3 s following inhalation, but that following exhalation i*f*_H_ decreased by an average of 42.6%. Thus, if *Ex* did not further reduce i*f*_H_ there would not have been a further reduction.

Although these results are preliminary, we show that the *Ex* and *In* phases of respiration result in rapid and opposite *f*_H_ responses. Thus, after breathing, the tachycardia followed by a slowly declining i*f*_H_, may be a compound effect of the rapid inhalation and/or lung inflation resulting in transient vagal withdrawal or sympathetic activation, followed by the gradual extinction of this chronotropic stimulation. In addition, it has been shown that both *f*_R_ and VT alter the magnitude of the change in i*f*_H_ (Fahlman et al., 2019b; Cauture et al., 2019; Blawas et al., 2021a; Blawas et al., 2021b). For example, when varying the *f*_R_ from 2 breaths • min^−1^ to 6 breaths • min^−1^ the average resting i*f*_H_ increased by 40% (Cauture et al., 2019; Fahlman et al., 2020b; Blawas et al., 2021a; Blawas et al., 2021b). Consequently, this makes it difficult to assign a resting *f*_H_ that can be used to compare within and between species (Kooyman, 1985). In comparative physiology, resting *f*_H_ is an important measure as it provides a link between how cardiac function relates to energy use, e.g., allometric scaling (He et al., 2023). In this context, the resting *f*_H_ should be the value that represents no limitation to blood flow to support organs with O_2_ for aerobic metabolism (Kooyman, 1985). However, variation in respiratory effort (*f*_R_ and VT) likely causes large variation between species and studies. Thus, without accounting for the cardiorespiratory coupling, studies, such as allometric scaling and diving physiology, that use an estimated “resting” *f*_H_ rate from intermittent breathers may have inflated variance.

Cardiac variability is a well-known phenomenon in continuous breathing mammals, referred to as respiratory sinus arrhythmia (RSA), where the i*f*_H_ varies throughout the respiratory phase, with increase and decrease of i*f*_H_ related to the inspiration and exhalation phases (Ben-Tal et al., 2012). The cyclical variation is thought to improve gas exchange or to maintain blood gases while minimizing the work of breathing (Hirsch and Bishop, 1981; Ben-Tal et al., 2012; Mortola et al., 2015). We propose that the respiratory tachycardia that we report in intermittent breathing bottlenose dolphin serves a similar purpose. Following inhalation, the pulmonary PO_2_ and PCO_2_ are, respectively higher and lower, which enhances the diffusion rate of both gases with the blood in the pulmonary capillary. The increase in instantaneous cardiac output, through increase in i*f*_H_ and an increase in the instantaneous stroke volume, helps to enhance O_2_ uptake and CO_2_ removal (Fahlman et al., 2020b). Inspiration likely also results in a transient change in arterial blood pressure (Figure 1B), which then is seen as the sinusoidal variation in i*f*_H_ during the inter-breath interval (Larsen et al., 2010). The initial increase followed by a decrease in both *f*_H_ and stroke volume helps reduce cardiac work, while also enhancing gas exchange. This cardiorespiratory strategy may be especially important as dolphins return to the surface following a dive, when reducing the recovery time at the surface allows the dolphin to return to the foraging patch. Past data in marine mammals have shown that the duration of the surface interval is mainly driven by CO_2_ removal from the tissues and blood (Reed et al., 1994; Reed et al., 2000; Boutilier et al., 2001; Fahlman et al., 2008) and elevated cardiac output helps transport CO_2_ to the lungs and enhances gas exchange. Thus, this respiratory tachycardia has a dual effect to reduce the surface interval.

In summary, in the current study we show that cardiorespiratory coupling in the bottlenose dolphin results in a respiratory tachycardia associated with the inhalation. When we separated the two phases of breathing, we observed that inhalation results in tachycardia similar to what we observed during a normal breathing cycle, while exhalation reduces i*f*_H_. The sinusoidal variation in i*f*_H_ seen throughout the inter-breath interval is likely the effect variation in arterial blood pressure (Figure 1B), which in turn alters i*f*_H_. We propose that the cardiorespiratory coupling reported in the bottlenose dolphin, which results in a temporary increase in cardiac output, improves gas exchange and reduces recovery time during a surface interval following a dive.

## Data Availability

The raw data supporting the conclusion of this article will be made available by the authors, without undue reservation.
